# Association between UGT1A1*28 Polymorphisms and Clinical Outcomes of Irinotecan-Based Chemotherapies in Colorectal Cancer: A Meta-Analysis in Caucasians

**DOI:** 10.1371/journal.pone.0058489

**Published:** 2013-03-14

**Authors:** Xiang Liu, Dangxiao Cheng, Qin Kuang, Geoffrey Liu, Wei Xu

**Affiliations:** 1 Ontario Cancer Institute, Toronto, Ontario, Canada; 2 School of Laboratory Medicine, Hubei University of Chinese Medicine, Wuhan, China; 3 Division of Medical Oncology and Hematology, Princess Margaret Hospital, University Health Network, University of Toronto, Toronto, Ontario, Canada; 4 Dalla Lana School of Public Health, University of Toronto, Toronto, Ontario, Canada; 5 Department of Biostatistics, Princess Margaret Hospital, University Health Network, University of Toronto, Toronto, Ontario, Canada; Sudbury Regional Hospital, Canada

## Abstract

**Background:**

Whether UGT1A1*28 genotype is associated with clinical outcomes of irinotecan (IRI)-based chemotherapy in Colorectal cancer (CRC) is an important gap in existing knowledge to inform clinical utility. Published data on the association between UGT1A1*28 gene polymorphisms and clinical outcomes of IRI-based chemotherapy in CRC were inconsistent.

**Methodology/Principal Findings:**

Literature retrieval, trials selection and assessment, data collection, and statistical analysis were performed according to the PRISMA guidelines. Primary outcomes included therapeutic response (TR), progression-free survival (PFS) and overall survival (OS). We calculated odds ratios (OR) and hazard ratios (HR) with 95% confidence intervals (CI). Twelve clinical trials were included. No statistical heterogeneity was detected in analyses of all studies and for each subgroup. Differences in TR, PFS and OS for any genotype comparison, UGT1A1*28/*28 versus (vs) UGT1A1*1/*1 (homozygous model), UGT1A1*1/*28 vs UGT1A1*1/*1 (heterozygous model), and UGT1A1*28/*28 vs all others (recessive model, only for TR) were not statistically significant. IRI dose also did not impact upon TR and PFS differences between UGT1A1 genotype groups. A statistically significant increase in the hazard of death was found in Low IRI subgroup of the homozygous model (HR = 1.48, 95% CI = 1.06–2.07; P = 0.02). The UGT1A1*28 allele was associated with a trend of increase in the hazard of death in two models (homozygous model: HR = 1.22, 95% CI = 0.99–1.51; heterozygous model: HR = 1.13, 95% CI = 0.96–1.32). These latter findings were driven primarily by one single large study (Shulman et al. 2011).

**Conclusions/Significance:**

UGT1A1*28 polymorphism cannot be considered as a reliable predictor of TR and PFS in CRC patients treated with IRI-based chemotherapy. The OS relationship with UGT1A1*28 in the patients with lower-dose IRI chemotherapy requires further validation.

## Introduction

Colorectal cancer (CRC) is the second leading cause of cancer-related death, and the most common cancer in the United States with 148,810 new cases and 49,960 deaths during 2008 alone [1]. Irinotecan (IRI) is one of the most effective chemotherapeutic agents in the treatment of CRC [2], [3]. At least 15% of individuals with new CRC are candidates for IRI therapy [1], [4].

IRI efficacy is dependent on activation by carboxyesterases to form the active metabolite 7-ethyl-10-hydroxycamptothecin (SN-38), which is a potent poison of topoisomerase I that interrupts DNA replication in cancer cells, resulting in cell death [5], [6], [7]. The major route of SN-38 elimination is via glucuronidation by the uridine diphosphate glucuronosyltransferase (UGT) 1A1, an essential enzyme involved in the complex metabolism of IRI [5]. UGT1A1*28 is a common allele with seven TA repeats in the promoter of UGT1A1 compared with the wild-type allele (UGT1A1*1) with six repeats [6], [7], [8]. A seven-repeat allele is associated with decreased gene transcription and expression of UGT1A1 and reduced enzyme activity, which lead to higher or more prolonged exposure of SN-38, the active form of IRI [8], [9].

Given that the UGT1A1 *28 variant influences IRI metabolism through enhanced exposure of its active metabolite SN-38, it is pharmacologically plausible that the UGT1A1*28 allele may be associated with the therapeutic efficacy of IRI in addition to the risk of adverse effects [10], [11]. Researchers have investigated the efficacy of IRI in CRC patients bearing different UGT1A1*28 genotypes [12], [13], [14], [15], [16], [17], [18], [19], [20], [21], [22], [23], [24], [25], [26], [27], [28], [29], [30], [31]. However, results are both conflicting and difficult to interpret because of small sample sizes and associated poor statistical power. Although a recent meta-analysis was performed to analyze the difference in therapeutic response (TR) between IRI-administered cancer patients with different UGT1A1*28 genotypes [10], it only provided data on TR, a surrogate for the most important outcome: survival, and included studies of different cancers rather than completely focusing on patients with CRC. This meta-analysis will therefore assess effects of UGT1A1*28 polymorphism on the efficacy of IRI-based chemotherapy, not only including TR but also survival. Moreover, it focuses on CRC alone, which will allow an assessment of uniform regimens tied to a single clinical disease site. In addition, two more recent publications on CRC are included [12], [13].

## Materials and Methods

### Retrieval of Published Studies

A comprehensive search of the PubMed and EMBASE databases was conducted from its inception through to July 2012 with the following search terms ‘irinotecan’, ‘UGT1A1’, ‘UGT1A1 polymorphism’, ‘UGT1A1*28’, ‘colorectal cancer’, ‘chemotherapy’, ‘response’, ‘progression-free survival (PFS)’, and ‘overall survival (OS)’. Furthermore, we screened titles and abstracts to identify relevant studies. Studies in abstract form or meeting reports, without publication of the full paper, were excluded.

The UGT1A1*28 polymorphism is relatively rare in Asian populations and the prevalence of homozygous UGT1A1*28 genotype is significantly greater in Caucasians than in Asian populations [32], [33], [34]. To reduce the heterogeneity among the analyzed studies, only studies involving mainly Caucasians populations were included in this meta-analysis.

### Inclusion and Exclusion Criteria

**Figure 1:**
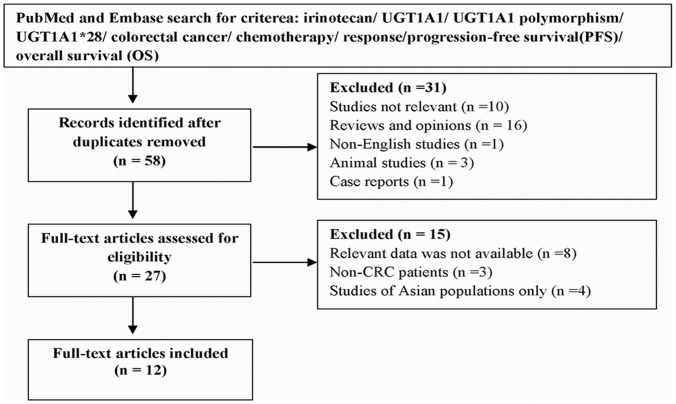
Flow diagram for study selection in meta-analysis.

Studies were included in the meta-analysis if (1) they were clinical trials or well characterized observational datasets, (2) they explored the association between UGT1A1*28 and clinical outcomes of IRI-based chemotherapy in patients with CRC, (3) there were sufficient data for TR (defined as a complete or partial response, using the WHO criteria [35] or the Response Evaluation Criteria in Solid Tumors criteria (RECIST) [36]) or PFS/OS data were provided, and (4) they were published in English. Exclusion criteria were as follows: (1) case reports, (2) reviews and opinions, (3) allele frequency studies, (4) studies not involving CRC patients, (5) studies where outcome data were not presented in detail or which had not provided enough information to calculate relevant data, and (6) studies conducted only in non-Caucasian populations. When different publications with overlapping subjects were considered eligible, we only included the one with larger numbers of patients. Figure 1 summarizes the search methods, inclusion and exclusion steps.

### Data Extraction

The following information was extracted from each eligible included publication: first author’s name, year of publication, country, primary race of patients, phase of clinical trial, number of patient, gender distribution, age (median or mean), source of population, polymorphism detection method, IRI dose, chemotherapy regimens, study design, response criteria, line of chemotherapy, and genotype data.

Two or three different IRI-containing regimens were administered to patients in some studies [15], [20]. When possible, we analyzed the patients treated with each regimen as separate samples. Patients treated with different regimens were analyzed as a single study only if separate data was not available. Sample sizes abstracted reflect the total number of patients who received IRI, as some trials also included non-IRI treatment arm.

### Statistical Analysis

PRISMA guidelines were followed (showed in Checklist S1) [37]. Odds ratios (OR) were used to estimate the association between UGT1A1*28 and TR. The OR was computed from the number of patients with and without TR after IRI-based chemotherapy. We evaluated PFS and OS based on pooled Cox proportional hazard ratios (HR) and 95% confidence intervals (CI) using published methods [38] because a meta-analysis of summary results is statistically as efficient as a joint analysis of individual participant data [39]. Between-study heterogeneity was assessed using the Cochran’s Chi-Squared test and the inconsistency index I^2^, with a significance level of P<0.05. We performed initial analyses with a fixed-effect model and confirmatory analyses with a random-effects model, if there was potential heterogeneity. We assessed potential publication bias by using a funnel plot and Egger’s test [40]. For meta-analysis that failed the Egger’s test (P<0.05), a trim and fill method was used to adjust for publication bias [41]. All statistical analysis was performed using Review Manager (v5.0; Oxford, England) and Stata software (Stata Corporation, Texas).

For TR, we compared the following: UGT1A1*28/*28 versus (vs) UGT1A1*1/*1 (homozygous model), UGT1A1*1/*28 vs UGT1A1*1/*1 (heterozygous model) and UGT1A1*28/*28 vs all others (recessive model). Two models (homozygous and heterozygous model) were examined in the analysis of PFS and OS. To assess the influence of IRI dose on the association between UGT1A1*28 and clinical outcomes, we carried out stratified analyses based on different IRI doses. In dose intensity analysis, 150 mg/m^2^ of IRI dose was set as the cutoff value between medium/high (High IRI) and low dose (Low IRI). In some studies [14], [20], [22], [23], the patients received different IRI doses at different time points and only combined data were available. The average dose was calculated to classify these studies.

## Results

### Characteristics of the Studies

Figure 1 shows the process of study selection. In total, 27 full-text studies were fully reviewed. Of these, five did not provide sufficient individuals’ genotype data [24], [25], [26], [28], [29]. Three only provided a summary description of their results in the text [27], [30], [31]. Four analyzed only Asian populations and were excluded [42], [43], [44], [45]. Three combined CRC patients with those that had other cancers [46], [47], [48]. Thus, only 12 studies were eligible for inclusion in our meta-analysis.

Characteristics of the included studies are summarized in Table 1. Methodologic components of study designs may be critically important to understand the meta-analyses results [49]; thus we utilize a modified set of criteria to report methodological issues and quality of the studies [50]. The criteria assessed study design, polymorphism detection method, combination regimens, Line of therapy, and grading systems for response (Table 1).

**Table 1:** Characteristics and methodological quality of studies included in meta analysis.

Study	Country, Races^a^	Phase of clinical trial	No.of patient (male%)	Age^b^	Population Source^c^	Mutation detection methods^d^	regimen^e^	Irinotecan dose (mg/m^2^)/schedule	Response criteria^f^	Line of regimens	Study design^g^	clinical outcomes^h^
Lamas 2012 [12]	Spain, U	U	100(63.4)	67	U	SPR	FOLFIRI	180/biweekly	RECIST	First and second line	R	TR, PFS^i^
Shulman 2011 [13]	Israel, C	I	329(48.0)	63	M	SPR	TEGAFIRI, XELIRI, FOLFIRI, IFL	U	/	U	R	OS
Martinez 2010 [14]	Spain, C	III	149(U)	U	M	Sequencing	FOLFIRI, FUIRI	80/weekly or180/biweekly	RECIST	First line	R	TR, OSi
McLeod 2010 [15]	USA/UK/Canada, mainly C	III	212(U)	61	M	PYRS	IFL, IROX	100–125/weekly or 200/every 3 weeks	U	U	P	TR, PFS, OS
Boige 2010 [16]	France, U	III	199(57.0)	67.5	M	SPR	FOLFIRI	180/biweekly	WHO	Second and third line	P	TR, PFS, OS
Glimelius 2011 [17]	Sweden/UK/Norway, mainly C	III	136(U)	62	M	SPR	FLIRI, Lv5FU-IRI	180/biweekly	RECIST		R	TR, PFS, OS
Toffoli 2006 [18]	Italy, C	I	250(64.8)	61	M	PYRS	FOLFIRI, mFOLFIRI	180/biweekly	WHO	First line	P	TR, PFS, OS
Rouits 2008 [19]	France, U	U	44(69.3)	60	S	PYRS	mFOLFIRI	180/biweekly	RECIST	U	U	TR, PFS^i^
Kweekel 2008 [20]	Netherlands, C	III	218(62.8)	61	M	PYRS	CapeIRI, IRI	250 or 350/every3 weeks	RECIST	First and second line	R	TR
Ruzzo 2008 [21]	Italy, C	U	146(55.6)	61	M	SPR	FOLFIRI	180/biweekly	RECIST	First line	P	TR, PFS
Rhodes 2007 [22]	USA, mainly C	U	51(57.4)	56	M	Sequencing	FOLFIRI, mIFL	125 or 180/biweekly	U	First line	U	TR
Carlini 2005 [23]	USA, mainly C	II	62(55.0)	61	M	SPR	CapeIRI	100 or 125/weekly	RECIST	U	P	TR

a C, Caucasian; U, Unknown.

b median or mean age.

c S, Single centre; M, Multicentre.

d SPR, Sizing of PCR products; PYRS, Pyrosequencing; Sequencing, other DNA sequencing methods.

e IR(I), irinotecan; 5FU, 5-fluorouracil; CAPe, capecitabine; OX(A), oxaliplatin; LV, leucovorin; XEL, xeloda; TEGAF, uracil/tegafur/LV.

f RECIST, Response Evaluation Criteria in Solid Tumors.

g R, analysis was planned retrospectively; P, analysis was planned prospectively.

h TR, therapeutic response; PFS, progression-free survival; OS, overall survival.

i These data were not available.

Of the 12 studies, three did not clearly report the race of the participants [12], [16], [19], but they were conducted in Europe or America. Because the UGT1A1*28 allele frequencies were similar to Caucasians, these three were classified with Caucasian studies. The study by Shulman et al [13] was included in the Low IRI subgroup based on the authors’ own comments. The results of meta-analysis are summarized in Table 2.

**Table 2:** The association between UGT1A1*/28 polymorphisms and therapeutic response, progression-free survival and overall survival.

Compared genotype	Group	Therapeutic response				Progression-free survival				Overall survival			
		Study (cases)	Fixed effect	Random effect	P_het_^a^	Study (cases)	Fixed effect	Random effect	P_het_^a^	Study (cases)	Fixed effect	Random effect	P_het_^a^
*28/*28 vs. *1/*1	**All**	11 (871)	1.09 [0.74,1.60]	1.09 [0.63,1.88]	0.13	5 (449)	0.86 [0.72,1.04]	0.90 [0.70,1.17]	0.18	5 (551)	1.22 [0.99,1.51]	1.22 [0.98,1.51]	0.39
	High IRI	8 (683)	1.13 [0.72,1.78]	1.10 [0.54,2.24]	0.09	5 (394)	0.82 [0.68,1.00]	0.82 [0.68,1.00]	0.45	4 (320)	1.09 [0.83,1.42]	1.09 [0.83,1.42]	0.49
	Low IRI	4 (188)	0.98 [0.46,2.08]	0.94 [0.39,2.26]	0.33	1 (55)	1.74 [0.85,3.56]	1.74 [0.85,3.56]	/	2 (231)	1.48 [1.06,2.07]	1.48 [1.06,2.07]	0.36
*1/*28 vs. 1/*1	**All**	11 (1390)	1.00 [0.80,1.26]	1.01 [0.80,1.26]	0.65	5 (734)	1.00 [0.86,1.17]	0.96 [0.77,1.19]	0.14	5 (893)	1.13 [0.96,1.32]	1.13 [0.96,1.32]	0.39
	High IRI	8 (1064)	0.96 [0.74,1.24]	0.96 [0.74,1.25]	0.59	5 (636)	1.00 [0.85,1.18]	0.93 [0.72,1.21]	0.08	4 (505)	1.10 [0.90,1.33]	1.10 [0.87,1.40]	0.22
	Low IRI	4 (326)	1.14 [0.73,1.79]	1.15 [0.73,1.81]	0.46	1 (98)	1.02 [0.65,1.60]	1.02 [0.65,1.60]	/	2 (388)	1.18 [0.91,1.53]	1.18 [0.91,1.53]	0.44
*28/*28 vs. *1/*28 or *1/*1	**All**	11 (1529)	1.08 [0.74,1.57]	1.10 [0.67,1.79]	0.20								
	High IRI	8 (1168)	1.16 [0.75,1.79]	1.16 [0.62,2.20]	0.15								
	Low IRI	4 (361)	0.89 [0.43,1.82]	0.83 [0.39,1.78]	0.42								

P_het_^a^ : P values for the between-study heterogeneity.

### Association between UGT1A1*28 and TR

**Figure 2:**
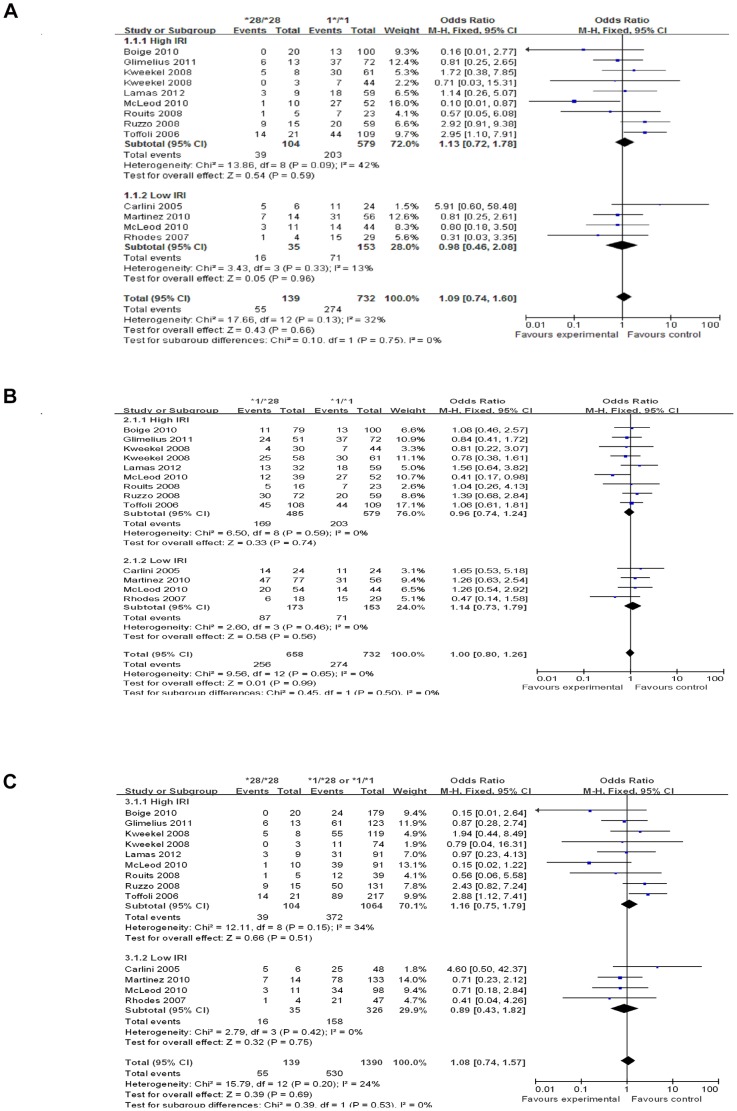
Forest plots of three comparisons; outcome: therapeutic response. 2A: *28/*28 versus *1/*1; 2B: *1/*28 versus *1/*1; 2C: *28/*28 versus *1/*28 or *1/*1.

Analysis of pooled data from all samples indicated that UGT1A1*28 allele was not associated with TR in CRC. In the homozygous model, the OR was 1.09 (95% CI = 0.74–1.60; P = 0.66) (Figure 2–2A). For UGT1A1*1/*28 vs UGT1A1*1/*1, the OR was 1.00 (95% CI = 0.80–1.26; P = 0.70) (Figure 2–2B). The recessive comparison had an OR of 1.08 (95% CI = 0.80–1.25; P = 0.69) (Figure 2–2C). Stratified analysis by IRI dose showed that the differences in TR between genotype groups were not statistically significant for any of the IRI dose levels (Table 2, Figure 2). The heterogeneity across all studies was not statistically significant for any model. I^2^ values were 32 (P = 0.13), 0 (P = 0.65) and 24% (P = 0.20) respectively for homozygous, heterozygous and recessive models (Table 2). No publication bias was detected by either the funnel plot (Figure S1) or Egger’s tests (P>0.05, each comparison).

### Association between UGT1A1*28 and PFS

**Figure 3:**
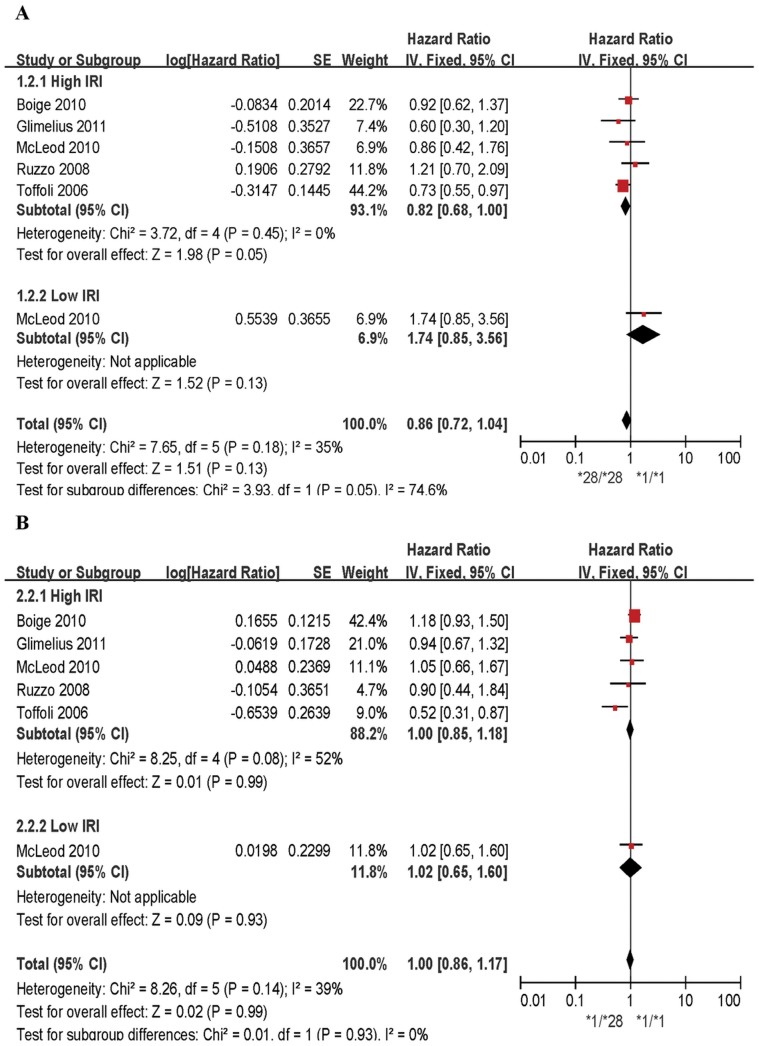
Forest plots of two comparisons, outcome: progression-free survival. 3A: *28/*28 versus *1/*1; 3B: *1/*28 versus *1/*1.

Pooled data from all samples for two genotype comparisons indicated that the UGT1A1*28 allele was not associated with a significant decrease of hazard for PFS in CRC (Figure 3 and Table 2). In homozygous and heterozygous models, the HRs were 0.86 (95% CI = 0.72–1.04; P = 0.18 for heterogeneity, I^2^ = 35%) and 1.00 (95% CI = 0.86–1.17; P = 0.14 for heterogeneity, I^2^ = 39%), respectively. Subgroup analyses based on IRI dose did not show any significant difference in terms of the association between UGT1A1*28 genotype and the hazard of PFS. There was no evidence of publication bias given the symmetrical distributions of funnel plots (Figure S2) and Egger’s tests (P = 0.28 and 0.14).

### Association between UGT1A1*28 and OS

**Figure 4:**
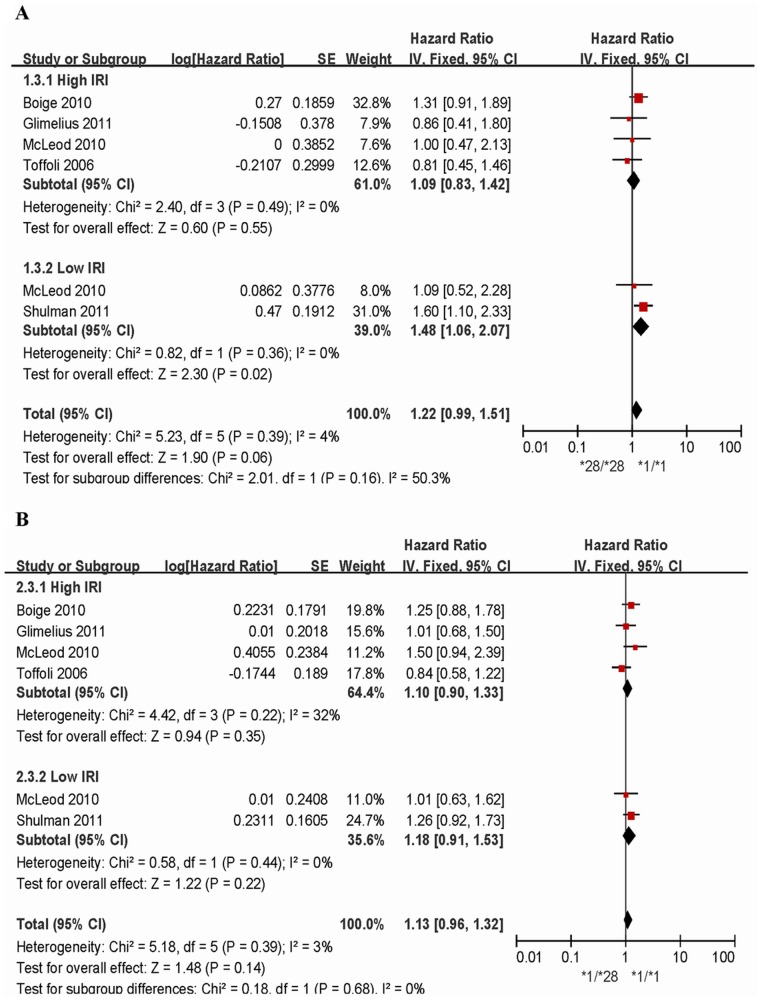
Forest plots of two comparisons, outcome: overall survival. 4A: *28/*28 versus *1/*1; 4B: *1/*28 versus *1/*1.

Five studies involving 551 patients were analyzed for the homozygous model and five studies (893 cases) for heterozygous model. The UGT1A1*28 allele was associated with a non-significant increase in the hazard of death in two models (homozygous model: HR = 1.22, 95% CI = 0.99–1.51, P = 0.06; heterozygous model: HR = 1.13, 95% CI = 0.96–1.32, P = 0.14) (Figure 4 and Table 2). However, subgroup analysis found a statistically significant increase in the hazard of death in Low IRI subgroup for the homozygous model (HR = 1.48, 95% CI = 1.06–2.07; P = 0.02). No associations were seen in the heterozygous model. No publication bias was detected in the funnel plots (Figure S3) and Egger’s tests (P>0.05), and there was no heterogeneity in each model (I^2^<5%, P>0.05).

## Discussion

While the association between UGT1A1*28 and IRI-related toxicity has been extensively studied, data are limited regarding the potential impact of the UGT1A1*28 genotype on tumor responsiveness and patient survival following IRI therapy [10]. Published meta-analyses have demonstrated dose-dependent associations between UGT1A1*28 genotype and IRI-induced neutropenia or diarrhea [51], [52], [53]. The U.S. Food and Drug Administration in 2005 recommended that gene-related information be added to the drug product label and approved the diagnostic UGT1A1*28 test to identify homozygous patients advising a lower dose of IRI in these patients [54]. However, a reduction in dosage might also be associated with reduced tumor response and/or increased morbidity [55]. The Evaluation of Genomic Applications in Practice and Prevention (EGAPP) working group and some cost-effectiveness analyses have indicated that UGT1A1*28 genotyping will only be clinically useful if dosing IRI on basis of genotype improves the safety of IRI without compromising the efficacy of the therapy [55], [56], [57], [58], [59], [60]. Thus, whether UGT1A1*28 genotype is associated with clinical outcomes of IRI-based chemotherapy is an important gap in existing knowledge to inform clinical utility [55], [56].

A previous meta-analysis [10] of 12 studies (8 studies [14], [15], [16], [17], [18], [20], [21], [23] were included in our meta-analysis) was performed to analyze the difference in TR between IRI-administered cancer patients with different UGT1A1*28 genotypes. Results indicated that differences in TR for all genotype comparisons were not statistically significant. Subgroup analyses based on IRI dose and tumor type (CRC and lung cancer) did not show any significant difference in terms of the association between UGT1A1 genotype and TR. The present meta-analysis assessed the association of UGT1A1*28 polymorphisms with clinical outcomes of IRI-based chemotherapy in a single cancer site, CRC. In our meta-analyses, the differences in TR between the different UGT1A1*28 genotype patient groups also did not attain statistical significance. Also, in contrast to IRI-induced toxicities, there was no convincing evidence to suggest that the association between UGT1A1*28 genotype and TR is modified by IRI dose. This is consistent with the meta-analysis of Dias et al [10], where no association between UGT1A1*28 genotypes and IRI response was found in an analysis across various tumor types including CRC. Similar results were detected in association between UGT1A1*28 genotypes and PFS. However, the UGT1A1*28 allele showed significant or marginal association with poorer OS, especially in Low IRI subgroup of homozygous model.

Our OS results are in the opposite direction of our original hypothesis. Possible explanations for why OS could be lower in patients carrying the UGT1A1*28 allele include suboptimal treatment due to the severity of adverse effects and the decreased dose intensity resulting from frequent dose reduction or treatment delay [13], [59]. These two parameters are intrinsically correlated but not necessarily consistent with one another [61]. OS is defined as the time from randomization to death caused by any reason and represents the gold standard metric for establishing efficacy. This typically requires phase III trials of large sample size with lengthy follow-up. TR and PFS as the alternative end points for OS occur earlier and can evaluate the effect of an intervention faster, at less cost with fewer trial subjects [62]. However, Prediction of TR and PFS are more complicated because other factors, such as tumor-related factors, environmental factors, and patient’s characteristics, should be considered [59]. Hence, TR and PFS may correlate with a real clinical endpoint (OS) but do not necessarily have a guaranteed relationship. That a reduction in IRI dosage among UGT1A1*28*28 patients with CRC may reduce the long-term survival (OS), but not influence on TR and FPS is intriguing; however, an underlying mechanism needs to be clarified.

The study conducted by Shulman et al was included in this meta-analysis, which may have driven the OS findings due to its large sample size (329 cases). This study suffered in particular from an unspecified IRI dosage and thus was only marginally accepted based on its methodology. An additional meta-analysis excluding the study showed that the results of HRs were 1.09 (homozygous model) and 1.08 (heterozygous model), respectively, which were slightly different from the HRs in the overall estimate of 1.22 and 1.13. However, the analysis failed to reach a statistical significance because of the insufficient power with the small sample size in this meta-analysis (only four studies involving 222 patients were analyzed for the homozygous model and 564 cases for heterozygous model). Thus, while our OS relationship is intriguing, much more validation is needed.

In our meta-analysis, three parameters (TR, PFS and OS) were used to assess the influence of UGT1A1*28 polymorphism on clinical outcomes. This presents a more comprehensive assessment than a single parameter as a prior meta-analysis had performed [61]. Moreover, our study only focused on CRC, reducing the potential heterogeneity across the studies. In addition, we paid attention to methodological components of study designs in the literature. The certain items such as study design, polymorphism detection method, combination regimens, Line of therapy, and grading systems for response, are reflective of methodological and reporting quality of the studies. It is beneficial to analyze the heterogeneity in this meta-analysis and improve the chances to replicate initial significant findings in subsequent pharmacogenetic studies [50].

There are limitations of this analysis. Firstly, some studies were excluded from our analysis because of lack of individual genotype data [24], [25], [26], [27], [28], [29], [30], [31]; this could cause some bias in our estimates, but was unlikely to change our major conclusions, as these excluded studies showed no association between UGT1A1*28 polymorphism and either TR or PFS in Caucasians. Secondly, there is inherent heterogeneity to all meta-analyses. In the analyzed studies, there were differences in study design, the source of population, IRI dose, polymorphism detection method, response grade criteria, therapeutic regimens, line of therapy, and performance status of patients. Additionally, although the difference in distribution of stage at diagnosis across studies will contribute to the heterogeneity in our meta-analysis, we did not perform a stratified subgroup analysis on stage at diagnosis because only three studies [13], [18], [21] provided data on stage and none explored the association between UGT1A1*28 and clinical outcomes of different stage at diagnosis. Likewise, other stratified subgroup analyses such as on the localization of primary tumor (six studies reported the localization of primary tumor [12], [14], [17], [18], [19], [20], but they did not provided the separated outcome data) could not be conducted. Thus, we performed initial analyses using a fixed-effects model and confirmatory analyses using a random-effects model. Results were similar between these two methods. Thirdly, articles included in this meta–analysis were restricted to English publishing studies. Articles with potentially high-quality data in other languages were excluded because of anticipated difficulties in obtaining accurate medical translation.

Although meta-analysis can synthesize the results of multiple studies into a summary of results, it is different from a combined analysis which uses the full information of individual patient data and can provide more comprehensive assessment. Our meta-analysis was useful because the collection of detailed information of each clinical trial was impractical and in some cases, not allowable due to local ethics board concerns [63].

In summary, this meta-analysis provided modest evidence for the association between UGT1A1*28 polymorphism and OS of IRI-based chemotherapy in CRC. UGT1A1*28 polymorphism cannot be considered a reliable predictor of TR and PFS to IRI-based chemotherapy in patients with CRC. In contrast, the OS may be affected by UGT1A1*28 status; the UGT1A1*28*/28 patients with CRC have a worse OS after lower-dose IRI therapy. The clinical significance of this last finding requires replication and additional research. In particular, as IRI metabolism is complex, numerous genes in addition to UGT1A1 should be interrogated [27].

## Supporting Information

Figure S1 **Funnel plots of three comparisons, outcome: therapeutic response.** 1A: *28/*28 versus *1/*1; 1B: *1/*28 versus *1/*1; 1C:*28/*28 versus *1/*28 or *1/*1.
(TIF)

Figure S2 **Funnel plots of two comparisons, outcome: progression-free survival.** 2A: *28/*28 versus *1/*1; 2B: *1/*28 versus *1/*1.
(TIF)

Figure S3 **Funnel plots of two comparisons, outcome: overall survival.** 3A: *28/*28 versus *1*1; 3B: *1/*28 versus *1/*1.
(TIF)
