# Supplementary figures and images for: Association between UGT1A1*28 Polymorphisms and Clinical Outcomes of Irinotecan-Based Chemotherapies in Colorectal Cancer: A Meta-Analysis in Caucasians
Source: PLoS One. 2013 Mar 14;8(3):e58489. doi: 10.1371/journal.pone.0058489 (PMC3597733; doi:10.1371/journal.pone.0058489)

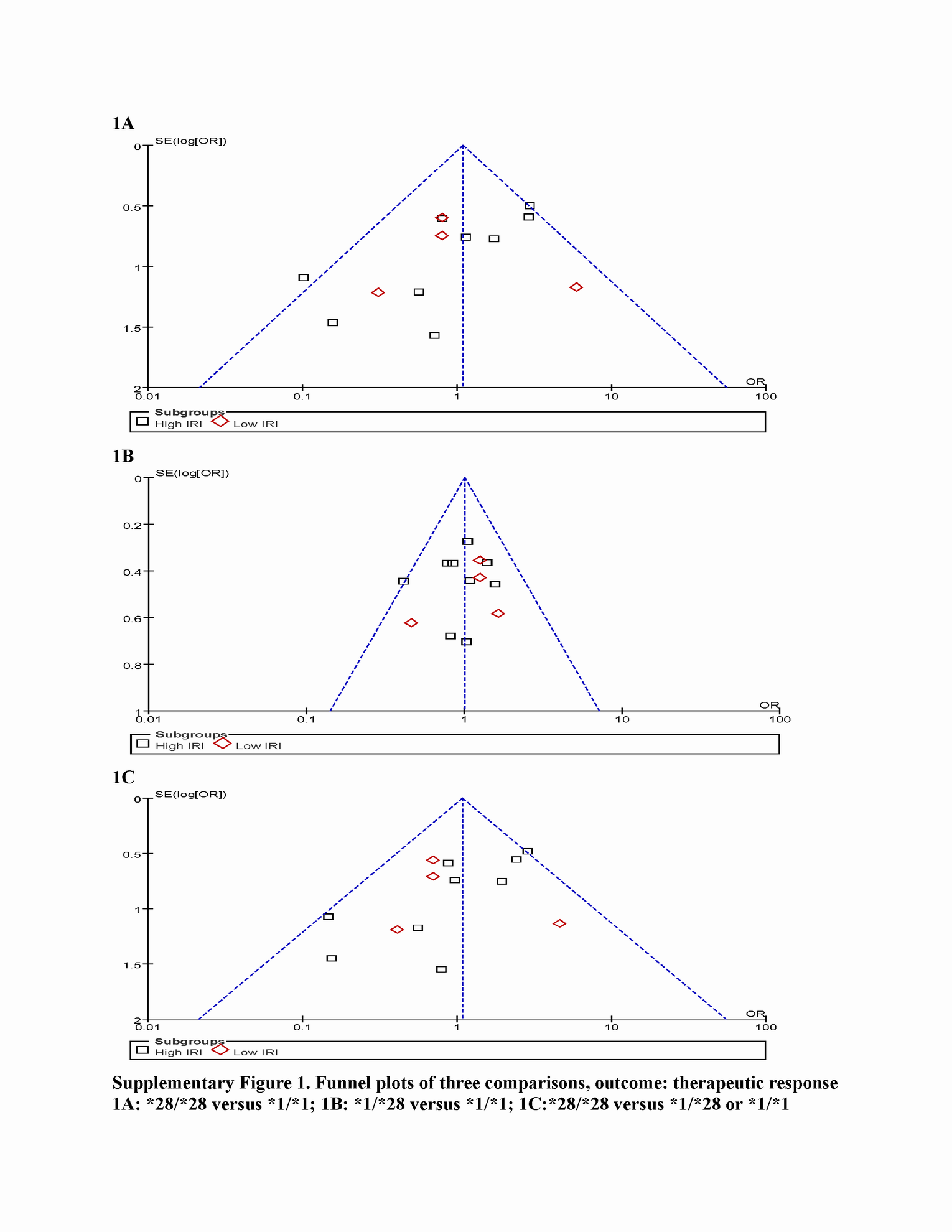

Supplement: Figure S1 — Funnel plots of three comparisons, outcome: therapeutic response. 1A: *28/*28 versus *1/*1; 1B: *1/*28 versus *1/*1; 1C:*28/*28 versus *1/*28 or *1/*1. (TIF) [file pone.0058489.s001.tif]

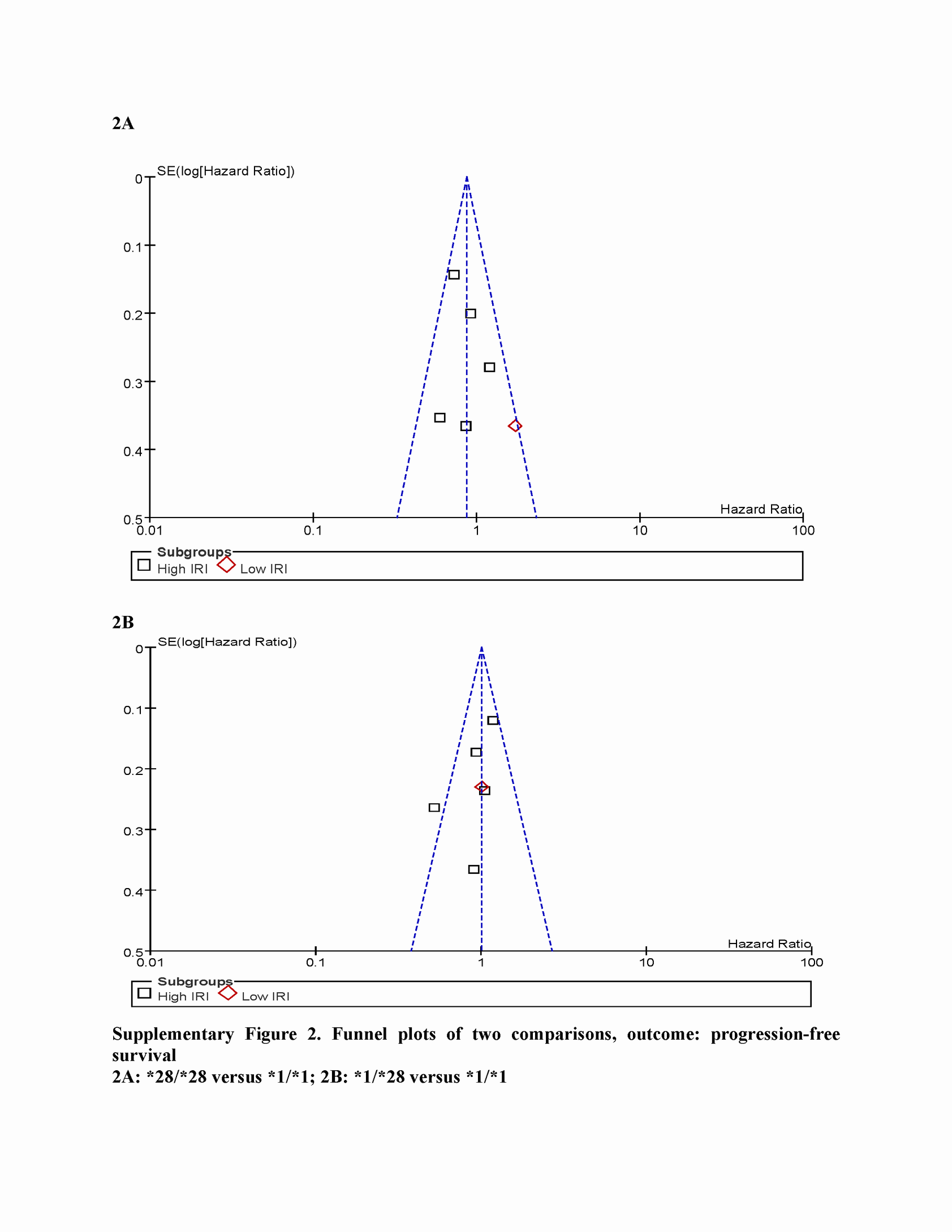

Supplement: Figure S2 — Funnel plots of two comparisons, outcome: progression-free survival. 2A: *28/*28 versus *1/*1; 2B: *1/*28 versus *1/*1. (TIF) [file pone.0058489.s002.tif]

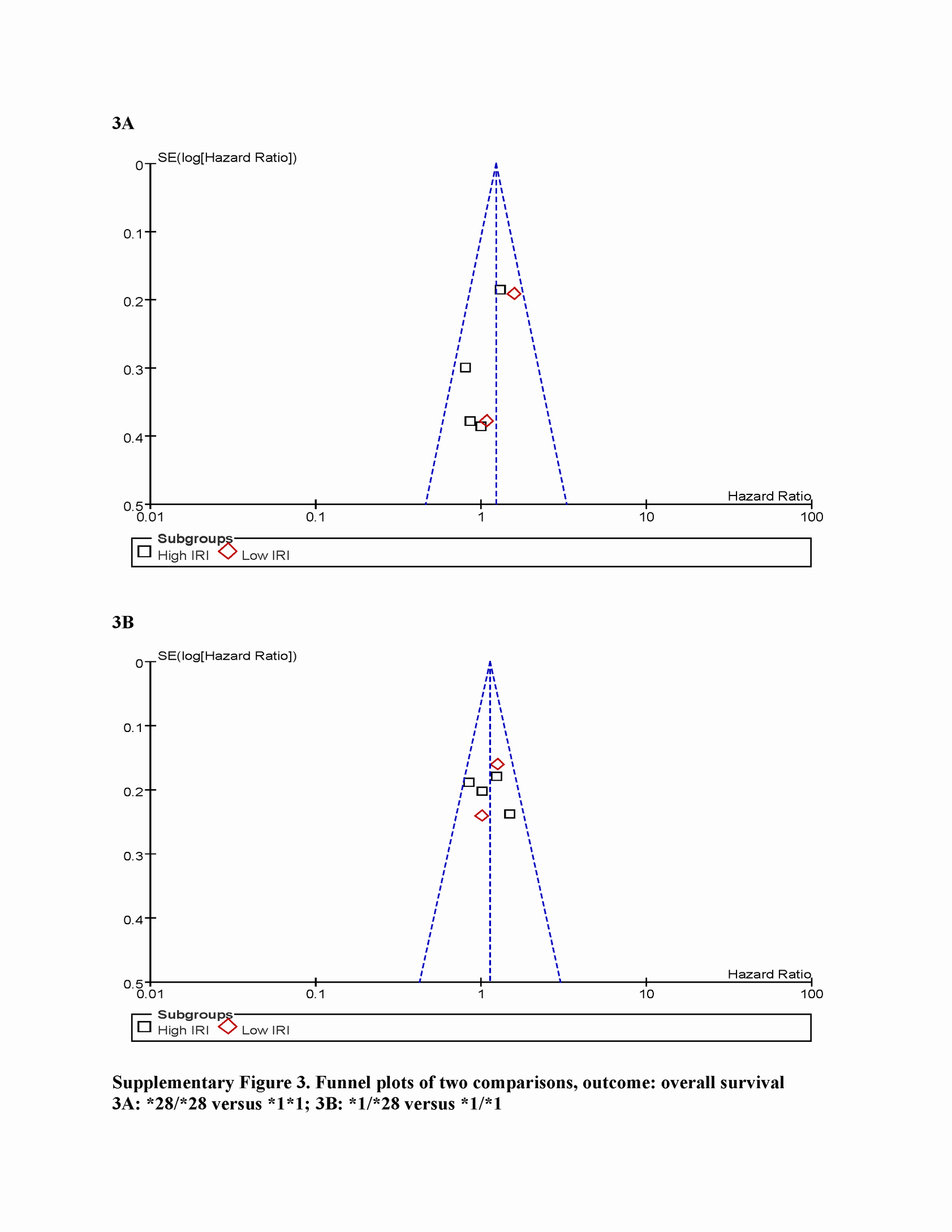

Supplement: Figure S3 — Funnel plots of two comparisons, outcome: overall survival. 3A: *28/*28 versus *1*1; 3B: *1/*28 versus *1/*1. (TIF) [file pone.0058489.s003.tif]
